# Dynamic Spatial Coding within the Dorsal Frontoparietal Network during a Visual Search Task

**DOI:** 10.1371/journal.pone.0003167

**Published:** 2008-09-09

**Authors:** Wieland H. Sommer, Antje Kraft, Sein Schmidt, Manuel C. Olma, Stephan A. Brandt

**Affiliations:** 1 Department of Neurology, Charité, Berlin Neuroimaging Center, Berlin, Germany; 2 Bernstein Center for Computational Neuroscience Berlin, Berlin, Germany; 3 Department of Clinical Radiology, University Hospital-Grosshadern, Ludwig-Maximilians University, Munich, Germany; Institut de la Vision, France

## Abstract

To what extent are the left and right visual hemifields spatially coded in the dorsal frontoparietal attention network? In many experiments with neglect patients, the left hemisphere shows a contralateral hemifield preference, whereas the right hemisphere represents both hemifields. This pattern of spatial coding is often used to explain the right-hemispheric dominance of lesions causing hemispatial neglect. However, pathophysiological mechanisms of hemispatial neglect are controversial because recent experiments on healthy subjects produced conflicting results regarding the spatial coding of visual hemifields. We used an fMRI paradigm that allowed us to distinguish two attentional subprocesses during a visual search task. Either within the left or right hemifield subjects first attended to stationary locations (spatial orienting) and then shifted their attentional focus to search for a target line. Dynamic changes in spatial coding of the left and right hemifields were observed within subregions of the dorsal front-parietal network: During stationary spatial orienting, we found the well-known spatial pattern described above, with a bilateral hemifield representation in the right hemisphere and a contralateral preference in the left hemisphere. However, during search, the right hemisphere had a contralateral preference and the left hemisphere equally represented both hemifields. This finding leads to novel perspectives regarding models of visuospatial attention and hemispatial neglect.

## Introduction

In the primate visual system, visual input from the retina reaches the primary visual cortex (V1) and is subsequently processed in a ventral and dorsal pathway. The ventral ‘perception’ pathway runs from the primary occipital cortex (V1) to the inferotemporal cortex and mainly processes foveal vision. However, the dorsal ‘action’ pathway runs from V1 to the frontoparietal network (FPN), which consists of regions in the posterior parietal cortex and the frontal eye fields (FEF). The FPN is known to represent the entire visual field and plays a critical role in spatial attention as well as action control, for example, goal-directed limb and eye movements [1].

There is a strong contralateral preference in the visual pathway from the retina to the primary visual cortex (V1). However, in higher level visual areas, patterns of contralateral preference are the subject of controversy. For higher level visual areas in the ventral pathway, a significant preference for contralateral stimuli was recently demonstrated for both hemispheres in the lateral occipital cortex and in object-selective and face-selective regions of the fusiform gyrus in both hemispheres [2], [3].

For the dorsal pathway consisting of the FPN, unequal representations of the visual hemifields have been intensely debated. According to the widely known model of Marsel Mesulam [4], frontoparietal areas of the left hemisphere have a contralateral preference and therefore represent mainly the right hemifield, whereas the corresponding areas of the right hemisphere represent both hemifields. This model attempts to explain why hemispatial neglect arises mainly after right hemispheric lesions and shows a deficit in attending to left-sided space. However, pathophysiological mechanisms of hemispatial neglect are controversially debated [5]–[8]. A recent model of Corbetta and Shulman (2002) [5] associates the dorsal frontoparietal network with top-down control of attention. The authors suggest that the activation in the dorsal FPN is predominantly bilateral for either visual field. In a subset of parietal areas the response might be spatially selective (stronger contralateral preference). In contradistinction, the ventral network should be involved in stimulus-driven attention and the authors propose that the anatomy of neglect better matches with the ventral attention system. They also mentioned that at present, it is not known whether the ventral network contains a spatial map that could direct attention to the location of unexpected events. In contrast, the detection deficits in neglect patients show a gradient across the visual field while the right TPJ responds equally well to stimuli in the contralateral and ipsilateral hemifield. Thus, Corbetta & Shulman [5] hypothesize that spatial precision might depend on the co-activation of the TPJ with the dorsal frontal network. They point out that topographical mapping of the TPJ and the dorsal network might indicate the relative role of each in directing attention to a location during exogenous orienting. Understanding the spatial coding in both networks will help to clarify the pathophysiology of neglect.

Recent fMRI studies using paradigms such as peripheral [9] and central detection tasks with peripheral stimulation [10] in healthy subjects describe unequal spatial representations within the dorsal FPN compatible with the Mesulam model. However, other visual tasks did not verify this unequal representation of space. Rather, a contralateral preference was found in both hemispheres during a delayed saccade task [11], a n-back working memory task [12] and a detection task in which the focus of attention systematically traversed the visual field [13].

Recently, it has been reported that contralateral preference in some cortical regions changes between finger pointing and saccade tasks [14]. In this paper, we address the question whether patterns of contralateral preference are dependent on different subprocesses of covert visuospatial attention, which in turn, might resolve previous controversial findings. Using a visual search task, we show that contralateral preference changes concomitantly between subprocesses of visuospatial attention in subregions of the dorsal pathway.

## Materials and Methods

### Subjects

All 25 participating subjects were strictly right-handed and had normal vision. Participants were students from the Humboldt-University Berlin and were compensated for participation in the study which was conducted in conformity with the Declaration of Helsinki and was approved by the ethics committee of the Charité Berlin. Written informed consent was obtained from all participants.

As in previous studies analyzing covert attention processes in visual search [15]–[17], it was important to make sure that subjects can perform the task without overt eye movements within a circular array of 7° visual angle. Each subject was tested in a behavioral experiment (240 trials) on the ability to perform the task correctly without eye movements. To ensure that subjects maintained proper fixation, eye-movements were recorded with the I-View-System (50 Hz) of SMI (Sensomotoric Instruments, Berlin-Teltow) applying the I-View 3.01.11 software. Subjects had to maintain fixation during the spatial orienting and visual search phase within 2° of the fixation cross. Eye data were analyzed with ILAB software [18]. Eleven of the test subjects (mean age = 25.3±2.3 years) fulfilled the criteria (eye-movements in less than 5% of all trials) and were subsequently tested in the fMRI experiment.

### Experimental paradigm

**Figure 1:**
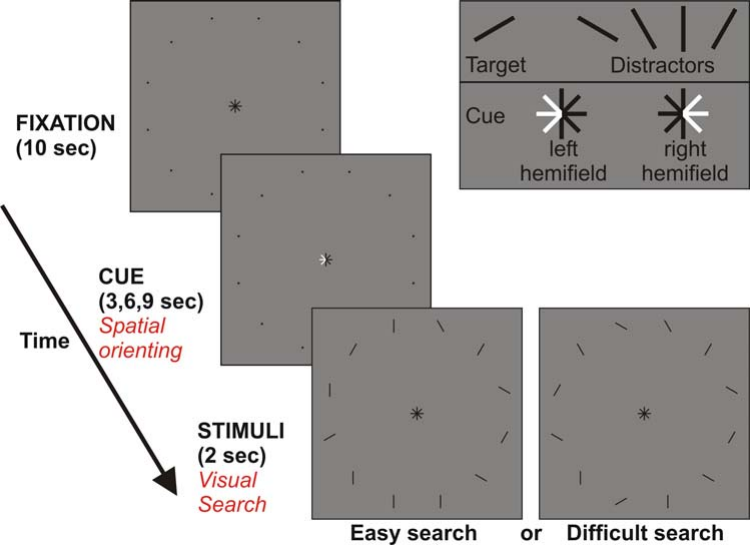
Paradigm separating spatial orienting (SO) and visual search (VS). After a fixation period, a central cue indicated the relevant hemifield for the next trial. When stimuli appeared, subjects had to search covertly for a target line among the 6 positions of the cued hemifield. Task difficulty was modified by the number of distractors with nonvertical orientations in the relevant hemifield (easy: 2; difficult: 5).

We utilized a novel event-related fMRI paradigm to investigate patterns of contralateral preference for two subprocesses of spatial attention in the same task (see Fig. 1). The subprocesses were either spatial orienting, in which the focus of attention remains stationary, or visual search, in which the focus shifts through the visual field. Our paradigm consisted of a circular array of 7° visual angle comprising 12 placeholders. Central fixation was maintained throughout the experiment. A trial started when one side of the central fixation symbol turned white, defining the relevant hemifield for the current trial. Subjects covertly allocated their attentional focus to the indicated hemifield (spatial orienting to six positions) for a variable period of time (3, 6 or 9 s). The variable delay served to prevent anticipatory responses. The placeholders allowed subjects to more precisely direct voluntary attentional shifts [19]–[21]. Subsequently, 12 black lines of 1° visual angle with different orientations appeared at the positions of the placeholders and a target line had to be detected among distractors within the previously cued hemifield (visual search at 6 positions). The target line had an angle of 30° counterclockwise to the horizontal meridian; the distractor lines had angles of 60°, 90° 120° and 150°, respectively (see Fig. 1). Task difficulty was modified by varying the number of lines that had nonvertical orientations in the relevant hemifield [22]. For easy conditions only two lines were nonvertical, whereas for difficult search conditions, 5 of the 6 lines in the cued hemifield had nonvertical orientations (see Fig 1). The same pattern of 6 lines was used throughout all trials in the non-cued hemifield. Equal numbers of easy and difficult trials were employed and the target was absent in 50% of all trials. Subjects had to respond with a target absent/present judgment as quickly and as accurately as possible by pressing buttons with their right index or right middle finger (randomized across subjects). Each experimental condition was repeated 60 times within 12 scanning runs in two sessions (12 trials with 3 s cueing interval, 24 trials with 6 s or 9 s cueing interval, respectively). Data from the 3 s cueing interval were only introduced to ensure that subjects paid attention during the whole cueing period. They were not further analyzed because of the delay in the BOLD response [15].

### Data acquisition

fMRI data were acquired in a 3 Tesla GE scanner using an 8-channel phased array coil. Stimuli were displayed by a LCD-projector and a custom-made lens on a small back-projection screen mounted in front of a standard head coil. Subjects viewed the screen via a mirror. A vacuum cushion inside the coil served to stabilize the subject's head and minimize head movements.

During the experimental blocks, we used a high resolution whole brain EPI sequence (voxel size 2 mm×2 mm×3.5 mm, TR = 3 s, TE = 60 ms, FA = 90°, 32 slices, 128×128 matrix). Each fMRI session included three preliminary saturation scans for T1 equilibration effects.

After three blocks, a 3D SPGR anatomical scan consisting of 222 slices was recorded to align the functional data on the high quality three-dimensional data set, which we acquired in an additional session on a Siemens 1.5 Tesla scanner using a T1-weighted sagittal Flash sequence (TR/TE = 38/5 ms, FA = 30°, voxel size = 1 mm^3^) with two acquisitions for excellent gray-white contrast for accurate segmentation and reconstruction of individual surface structures.

### Data analysis

fMRI data were analyzed using BrainVoyager QX (BrainInnovation, Maastricht, Netherlands). All anatomical and functional data were individually registered into a 3D stereotactic coordinate system [23]. Functional data preprocessing included slice time correction, motion correction, linear trend removal and high pass filtering of frequencies above 3 cycles per time course to remove slow drifts in fMRI signal. Blocks with motion exceeding 2 mm were excluded. For one subject, the data from three of twelve blocks had to be excluded; the data from another subject was excluded completely from analysis due to excessive motion in numerous blocks.

We segmented and reconstructed the surface of the white matter from the high resolution structural MRI images of each subject. Four ROIs of the FPN were predefined for each subject by anatomical landmarks: AIPS and PIPS (anterior and posterior intraparietal sulcus (IPS)), IPTO (IPS junction with the transverse occipital sulcus) [16], [17], [24] and the frontal eye field (FEF) [25]. Functional data were then realigned onto the high resolution T1-Flash images by using the anatomical information of the 3D SPGR anatomical scans.

**Figure 2:**
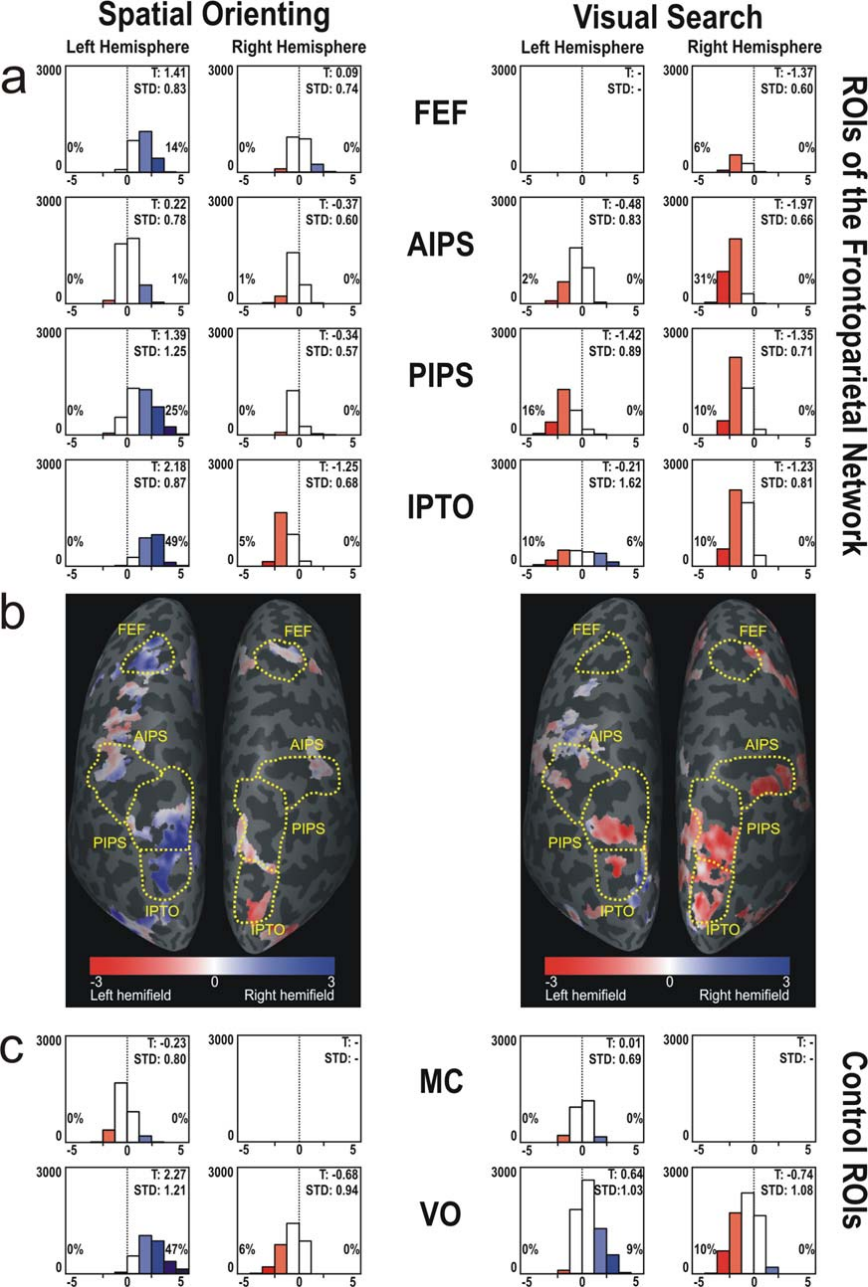
Contralateral preference of activated voxels during spatial orienting (SO) and visual search (VS). Contralateral preference visualized by t-tests for all voxels activated by SO and VS (p<0.005, RFX, cluster threshold: 50 mm^2^). Negative (red) and positive (blue) t-values indicate a preference for the left and right hemifield, respectively. Voxels with t-values<−2.26 or >2.26 show a significant contralateral preference (p<0.05) and are indicated by dark blue or red, respectively. White color indicates voxels involved in the process with no preference for either hemifield. (a) Histograms with t-values for all activated voxels within predefined ROIs of the dorsal FPN. The range of t-values represented by each bar is 1.13. The significance level (p<0.05) for contralateral preference to the left (−) or right (+) is indicated by black lines on the x-axis of each histogram. Additionally, the percentage of voxels with a significant contralateral preference for the left or right hemifield is shown on the appropriate side of the histograms. (b) Dorsal posterior view of the flattened left and right hemisphere with representation of t-values on the surface. (c) Histograms with t-values for all activated voxels within control ROIs MC (motor cortex) and VO (visual occipital). T: mean t-value of activated voxels; STD: standard deviation of t-values; Yellow dotted lines: predefined anatomical ROIs.

A random effects analysis (RFX, p<0.005, cluster threshold of 50 mm^2^) was performed on the regions activated by SO and VS periods, and they were separately marked on the surface of a single subject. It is important to note that these activated voxels were defined across both ipsilateral and contralateral stimulus presentations. For each activated voxel (1 voxel = 1 mm^3^) that corresponded to the activation on the surface (range from −1 to 3 mm), a t-test was performed separately for SO and VS, calculating whether the voxel responded more strongly to the left or the right hemifield condition [26]. These t-tests were based on the beta values of the voxel during the left or right hemifield condition, respectively. Resulting negative and positive t-values represented a preference for the left or right hemifield, respectively. T-values for each voxel were color-coded on the surface, with red for left hemifield preference and blue for right hemifield preference (see Fig. 2b). T-values around zero were coded by white and represented voxels that were activated by the subprocess of attention, but did not show a preference for either hemifield. Additionally, t-values were displayed in histograms, depicting the degree of contralateral preference within each ROI (see Fig. 2a).

Mean RTs, accuracy rate and response criterion differences (percentage of error, d' the measure of target detection sensitivity, c the measure of response criterion; [27]) were calculated separately for VS in the left and right hemifield and for easy and difficult feature search conditions, respectively. The response criterion is a measure of response bias (present or absent response-tendency). It is calculated by adding the z-scores of the hit-rate probability and of the false alarm rate probability, multiplied with – 0.5 [27].

Statistical data analyses were conducted with the SPSS software (Version 12.0). Mean RTs, accuracy rates and the response criterion were entered in two-way repeated measures ANOVAs with factors task difficulty and hemifield.

## Results

### Behavioral results

We calculated separate two-way ANOVAs with factors “task difficulty” (easy vs. difficult search) and “hemifield” (left vs. right hemifield) for RTs, percentage of error, d' and c, respectively (see Table 1). There was a significant main effect for the factor “task difficulty” for RTs [F(1,9) = 91.77; p<0.001], percentage of error [F(1,9) = 23.77; p<0.001], d' [F(1,9) = 23.36; p<0.001] but not for c [F(1,9) = 1.8; p>0.05]. However, none of these measures achieved a significant main effect for the factor “hemifield” with RT: [F(1,9) = 3.98; p>0.05], percentage of error [F(1,9) = 2.62; p>0.05], d' [F(1,9) = 2.10; p>0.05] and c [F(1,9) = 1.7; p>0.05]. There were no significant interactions between these two factors (RT: [F(1,9) = 0; p>0.05]; percentage of error [F(1,9) = 0.11; p>0.05]; d' [F(1,9) = 0.05; p>0.05]; c [F(1,9) = 1; p>0.05]).

**Table 1:** Summary of behavioral results.

Condition	RT (ms)	ER (%)	d'	c
Left hemifield	1229±59	4.45±0.66	3.51±0.14	0.18±0.07
Right hemifield	1207±56	5.52±0.99	3.41±0.17	0.29±0.07
Easy Search	1090±59	2.60±0.38	3.90±0.07	0.19±0.06
Difficult Search	1352±58	7.36±1.24	3.12±0.20	0.28±0.07
Easy - left hemifield	1101±60	2.20±0.67	3.76±0.15	0.11±0.08
Easy - right hemifield	1076±56	3.01±0.74	3.59±0.10	0.27±0.07
Difficult - left hemifield	1364±61	6.70±1.27	3.03±0.19	0.25±0.07
Difficult - right hemifield	1339±58	8.03±1.34	2.92±0.16	0.32±0.09

Reaction times (RT), error rates (ER), target detection sensitivity (d') and the response criterion (c) for visual search in the left and right hemifield, comparison of easy and difficult search conditions and comparison of the behavioral data of the left and right hemifield during easy and difficult search conditions. Data shown ± standard errors.

The response criterion showed positive values in all conditions, indicating that the frequency of missed targets was higher than of false alarms. The positive criterion corresponds to a “no-tendency” for the responses, which is a known phenomenon for visual search tasks [28]. In most visual search tasks, distractors are more common than targets, and therefore subjects adapt to a strategy to classify unclear stimuli rather as a distractor than as a target. This in turn leads to the observed “no-tendency” corresponding to positive values of the criterion.

### fMRI results

#### Spatial coding

During the SO and VS subprocesses, we found activation patterns that corresponded to the known FPN [17], [24]. Regarding the hemispheric representation of the hemifields, our analysis revealed that patterns of contralateral preference dynamically change between subprocesses of spatial attention within the same task. Mean t-values for activated voxels during the SO period indicated a pattern of contralateral preference according to Mesulam's model described above [4] (see Fig. 2a, left). In early visual areas (VO), both hemispheres showed a contralateral preference that was stronger in left VO (mean t-value T: 2.27) than in right VO (T: −0.68) (see Fig. 2c, left). Within the left FPN, positive mean t-values were observed in IPTO (T: 2.18), PIPS (T: 1.39) and the FEF (T: 1.41) which corresponded to a preference for the right hemifield. However, in AIPS of the left hemisphere, there was no clear contralateral preference (T: 0.22). In contradistinction, the right hemispheric ROIs of the FPN showed a preference for the left hemifield (corresponding to negative t-values) only in IPTO (T: −1.25), whereas in PIPS, AIPS and the FEF, t-values indicated a comparable representation of both hemifields that corresponded to t-values around zero (PIPS T: −0.34; AIPS T: −0.37; FEF T: 0.09).

During the VS period, early visual areas of both hemispheres showed a comparable, moderate preference for the contralateral hemifield (left VO (T: 0.64); right VO: (T: −0.74)) (see Fig. 2c, right). In the FPN, however, the pattern of contralateral preference was reversed to the pattern of SO. This time, right hemispheric ROIs preferentially processed the contralateral hemifield as indicated by its negative t-values (IPTO T: −1.23; PIPS T: −1.35; AIPS T: −1.97; FEF T: −1.37). In contrast, within the left hemisphere, ROIs of the FPN either showed an equal representation of both hemifields (IPTO T: −0.21; AIPS T: −0.48) or even an ipsilateral preference (PIPS T: −1.42) (see Fig. 2a, right).

**Figure 3:**
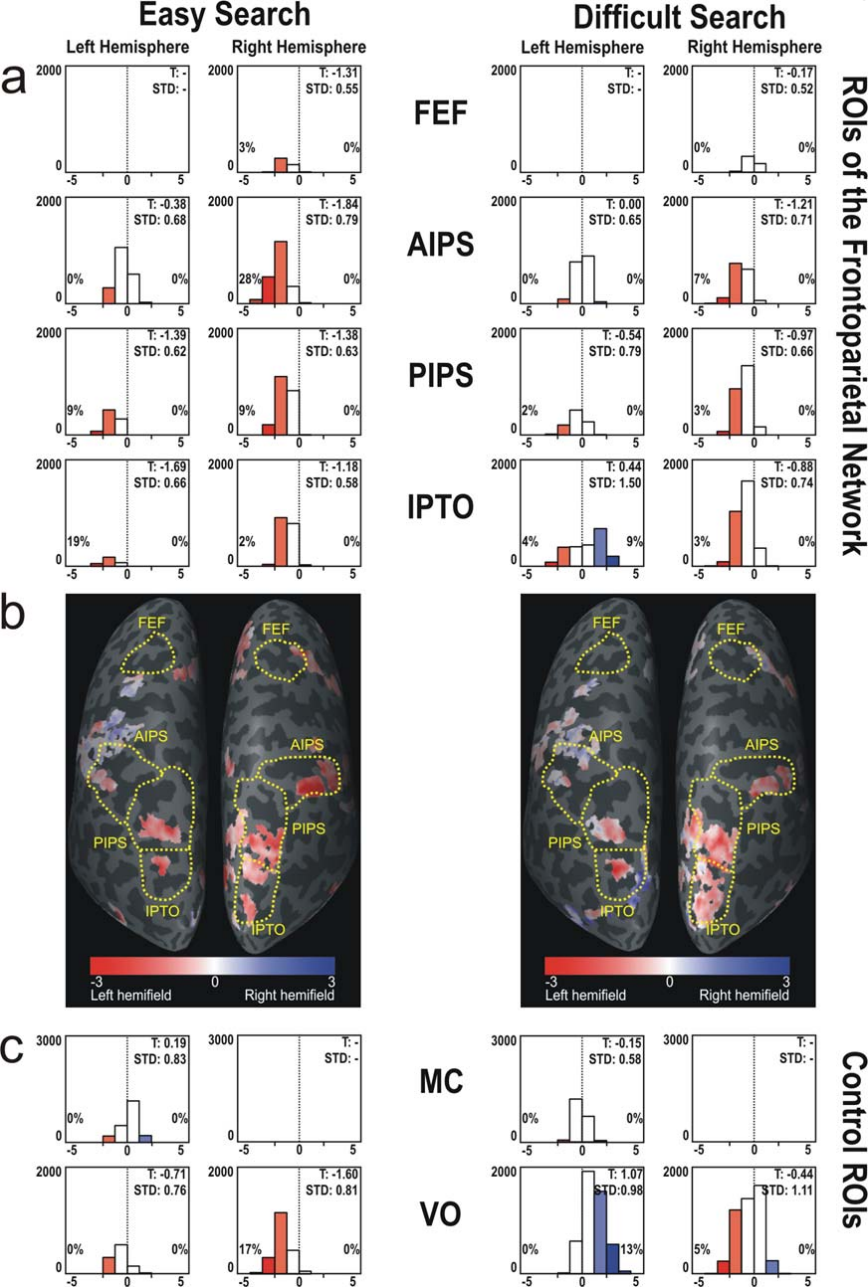
Contralateral preference of activated voxels during easy and difficult search conditions. Contralateral preference visualized by t-tests for all voxels activated by easy search and difficult search conditions (p<0.005, RFX, cluster threshold: 50 mm^2^). The color convention and the significance level for hemifield preference are described in Fig. 2. (a) Histograms with t-values for all activated voxels within predefined ROIs of the dorsal FPN. The range of t-values represented by each bar is 1.13. The significance level (p<0.05) for contralateral preference to the left (−) or right (+) is indicated by black lines on the x-axis of each histogram. As in Figure 2, the percentage of voxels with a significant contralateral preference for the left or right hemifield is given on the appropriate side of the histograms. (b) Dorsal posterior view of the flattened left and right hemisphere with representation of t-values on the surface. (c) Histograms with t-values for all activated voxels within control ROIs MC (motor cortex) and VO (visual occipital). T: mean t-value of activated voxels; STD: standard deviation of t-values; Yellow dotted lines: predefined anatomical ROIs.

The change of contralateral preference for SO and VS was evident both for easy and difficult search conditions (see Fig. 3). Furthermore, easy search condition showed even lower t-values within our predefined regions (p<0.01) and corresponded to a more ipsilateral tendency in the left hemisphere and to a higher degree of CP in the right hemisphere.

The control ROI of the left motor cortex revealed no hemifield specificity, neither during SO and VS, nor during easy or difficult search, respectively (see Fig. 2c and 3c).

#### Further results

Among the ROIs of the FPN, only IPTO showed a dependency of activated voxels on task difficulty (see Table 2). Additionally, activation in early visual areas (VO) was highly dependent on task difficulty. For VO, the total number of activated voxels is given here since VO was not anatomically predefined and therefore percentages of activated voxels could not be calculated. VO left (easy search: 1017 voxels; difficult search: 4711 voxels), VO right (easy search: 1980 voxels; difficult search: 4772 voxels). The remaining ROIs (PIPS, AIPS and FEF) did not show a comparable dependency on task difficulty.

**Table 2:** Activated voxels in the left and right ROIs of the FPN during spatial orienting (SO) and visual search (VS).

ROI	ROI size (mm)	SO (%)	VS (%)	easy VS (%)	difficult VS (%)
left FEF	3863	65	0	0	0
right FEF	4451	50	18	9	11
left AIPS	7437	56	44	26	24
right AIPS	5460	40	56	38	29
left PIPS	8719	48	29	10	11
right PIPS	5970	27	68	36	40
left IPTO	4997	41	39	6	42
right IPTO	5846	46	81	30	53
**Total left FPN**	**25016**	**52**	**31**	**12**	**19**
**Total right FPN**	**21727**	**40**	**58**	**30**	**34**

Anatomically defined ROI sizes (1 mm×1 mm×1 mm voxels); Percentage (%) of activated voxels during spatial orienting (SO) and distinct visual search (VS) conditions.

In addition, there was a slight difference in the lateralization of activated regions between these two subprocesses of spatial attention. Comparing the percentage of activated voxels among all voxels within the predefined ROI at the given significance level (RFX, p<0.005), we found a lateralization of the activation pattern into the left FPN ROIs during SO, especially for the subregions PIPS, AIPS and FEF (see Table 2 & Fig. 2). In contradistinction, during VS, the lateralization pattern was inversed: the activations were more strongly lateralized to the right FPN ROIs than to the left ones. This lateralization into the right hemisphere during VS was most prominent in IPTO, PIPS and the FEF. It was evident both for easy and difficult search conditions, but the lateralization into the right hemisphere was stronger during easy search.

Detailed numbers of voxels and percentages of activated voxels during SO and VS for all ROIs of the FPN are given in Table 2.

## Discussion

Our data revealed that the pattern of contralateral preference in the dorsal “action” pathway changes between different subprocesses of visuospatial attention. In fact, we observed the well-known pattern of spatial representation according to Mesulam's model during the subprocess SO [4]. Here, the left hemisphere had a strong preference for the contralateral field, while the right hemisphere processed both hemifields equally. In contrast, VS led to an inversed pattern of contralateral preference in which the right FPN showed a preference for the left hemifield. In the left hemisphere, however, regions along the IPS showed rather comparable representations of both hemifields. This change of pattern was observed both in easy and difficult search conditions, but was more prominent in easy search condition.

### Spatial coding

Our results indicate a difference between areas of the dorsal “action” pathway and the high-level areas of ventral “perception” pathway where stable preferences for contralateral stimuli were found, for example, in the object-selective and face-selective cortex [2]. It is important to note that the unequal hemifield representations in our study started to arise in regions along the IPS, while visual areas showed a comparable contralateral preference. This argues against a systematic bias of our task. Additionally, since our data were obtained within the same group of subjects and within the same experimental paradigm, the limited comparability of fMRI results due to interexperiment and intersubject variabilities is eliminated.

The disparity in visual field representations was also not explained by differences in behavioral results between the left and right hemifield; no significant differences had been found between visual search in the left and right hemifield, neither in reaction times nor in the accuracy rates. Furthermore, an overall increase in activation within both hemispheres under higher task demands was evident only in the visual areas and area IPTO, indicating a successful manipulation of task difficulty on a neural level [29].

It could be argued that during VS, the attentional focus was shifted within one hemifield in both ipsiversive and contraversive directions, while the initial shift of SO was only in one direction, the one of the cued hemifield. Furthermore, while left-right shifts take place in SO, up and down shifts are additionally needed during search. By this argumentation, the different patterns of contralateral preference during these subprocesses could at least partly be explained by the different directions of the attentional shifts. Furthermore, the higher degree of contralateral preference in the easy search condition could be explained in the same way by numerous shifts needed for target detection in the difficult search condition. However, previous experiments demonstrated that activations within the FPN depend more on the hemifield in which the attentional focus is located than on its direction [30]. Moreover, a recent study of Macaluso and Patria (2007) [31] showed that the axis of orientation of attentional shifts does not produce differences in brain activations. Therefore, this reasoning does not seem strong enough to fully explain the large differences of patterns found during SO and VS. But further research is necessary to analyze how the upper and lower visual fields are spatially coded within the hemispheres during spatial orienting compared to search.

Beside the dynamics of the attentional focus (stationary in SO, shifting in VS) the differential findings of spatial coding in SO, easy and difficult search conditions might result from a difference between endogenous (sustained) and exogenous (transient) attention processes [32]–[34]. In our study, SO was clearly guided by endogenous attention (central cue, long interval), which is strongly top-down driven [32]–[34]. In contrast, it is not so clear whether VS was implemented via endogenous or exogenous shifts of attention, or a mixture of both. While the observer can implement an endogenous task set to look for information in a certain manner, the saliency of stimulation will also guide the search [35]. Easy and difficult search conditions might affect whether the VS task is implemented exogenously or endogenously. In the case of the easy task, endogenous attention might not be necessary because the target may pop out, causing an exogenous, stimulus-driven shift of attention. In contradistinction, difficult VS seems to be a mixture of stimulus-driven exogenous and endogenous top-down-guided attention as the search array is too complex for purely stimulus-driven attention. The gradual change of contralateral preference from SO (endogenous) to difficult search (endogenous and exogenous) and easy search (exogenous) can be explained by this attentional dimension. Thereby, the lateralization of exogenous and endogenous attention has to be taken into account. The results of spatial coding match well with the change of lateralization during SO, difficult search and easy search condition. This will be discussed in more detail in the next section.

It is also important to note that the effects during VS reflect spatial attention mechanisms since stimuli were presented over both hemifields [3]. Thus, the resulting differences between the hemifields during VS cannot be ascribed to sensory stimulation differences between the hemifields. However, while SO exclusively measures the effect of attention, VS reflects the combined influence of sensory stimulation and spatial attention. As suggested by Hemond and colleagues [2], the relative contribution of these two factors might vary across processing stages. For instance in the present study, the FEFs were mainly activated during SO and showed only a small effect during target presentation. This is consistent with previous studies showing that the FEFs are mainly involved in large scale attention shifts and maintenance of attention [16], [36], [37].

Furthermore, in the present study, only VS required a motor response made with the right hand. Thus, it should be discussed whether spatial coding of the left and right hemifield can also be influenced by the side of response hand. Currently, there is no evidence in the literature that the response hand might influence spatial coding in visual areas or areas of the FPN [12], [13].

### Lateralization

In our data, SO showed an activation pattern slightly lateralized into the left hemisphere, while VS slightly more activated the right FPN. In Corbetta & Shulman's model [5], a strong asymmetry for the right hemisphere is only proposed for the ventral FPN. Otherwise, the authors also described that the activation in the dorsal FPN is predominantly bilateral for either visual hemifield, but in a subset of parietal areas, the response is spatially selective (stronger CP) and slightly right lateralized 8, 38, 39. Additionally, they mentioned that the dorsal FPN corresponds to the parietal and frontal cores of the Mesulam model. Our results of slight lateralization within the dorsal FPN are in line with these findings but for the first time demonstrated a direct change of lateralization within the same paradigm.

On the other hand, several alternative accounts have to be ruled out for the difference in lateralization between SO and VS:

It is unlikely that SO and VS differ in their local vs. global attention dimension. During SO we used placeholders to prevent subjects to attend the whole hemifield [15], [16], [19]–[21]. Irrespective of placeholders, it has been proposed that more global stimuli were preferentially processed in the right hemisphere [40]. From that perspective, one would expect a stronger lateralization during SO to the right hemisphere. Our findings are diametrically opposed to the global-local-hypothesis.

Further, SO and VS differ in the necessity of a perceptual decision. Perceptual decision by itself might require more left-lateralized resources [41], which is not in line with our results. A perceptual decision was only necessary during VS, where we found a slightly right-lateralized activation.

As described above, VS and SO periods differ in their necessity of a motor response. As the response was not counterbalanced across subjects it is impossible to ascertain whether the differences in lateralization are influenced by the right hand response to the search display. Reviewing the literature addressing lateralization in attentional subprocesses [17], [38], [39], [42], [43] revealed that only the study of Hopfinger and colleagues counterbalanced the hand of response across subjects. All other studies used the right hand across subjects. Only one response after target presentation was necessary in most of the studies. Slight lateralizations were reported for spatial orienting to the left hemisphere and for search to the right hemisphere. In the current study we found a lateralization to the right hemisphere during search, which is in line with the results of Donner et al. (2000) [17]. If the right response hand influenced the lateralization of activation, one would rather expect a bias of activation to the left hemisphere. Also, the left hemisphere has a general dominance for action [44]. In consequence, the unbalanced response hand doesn't seem to account for the slight differences in lateralization between SO and VS.

A difference between endogenous and exogenous attention processes could also account for the change of lateralization between SO and VS [32]–[34]. It is under discussion whether the same or distinct neural networks are involved in endogenous and exogenous attention processes [45]–[48]. According to the model of Corbetta & Shulman (2002) [5], a right lateralization should be observed if exogenous attention plays a crucial role. Hahn et al. (2006) also proposed, that top-down (i.e. endogenous) attention shows a stronger lateralization into the left hemisphere than bottom-up (i.e. exogenous) attention processes [42]. In line with these findings Gainotti (1996) [49] proposed that volitional orienting of attention is stronger lateralized to the left hemisphere (see also [45]). Consistent with these predictions, in the present study SO showed a slight lateralization to the left frontoparietal network while difficult and easy search showed a slight or stronger lateralization to the right frontoparietal network, respectively [38], [39], [42], [43].

### Spatial coding and lateralization

It is known that spatial and nonspatial functions overlap within the FPN [50]. This could also account for the change in spatial coding and lateralization as it was observed in our experiment. In both SO and VS, a bilateral network of regions with non-spatial functions is activated that does not show a pattern of contralateral preference and should be equally activated by both hemifields. For instance, in the present study, the left AIPS was equally activated for both hemifields during SO and VS. This is consistent with previous studies, suggesting that the left AIPS is predominantly involved in feature-based attention and object identification [16], [17], [51]. In contrast, regions concerned with spatial attention would show a contralateral preference in both the left and right hemispheres (e.g., area PIPS): SO is mainly processed by the left hemispheric network and VS by the right hemispheric network. For the former subprocess, this results in a higher degree of contralateral preference in the left hemisphere and for the latter, it explains the higher degree of contralateral preference in the right hemisphere. Additionally, it leads to the direct change of lateralization in our paradigm. In line with this finding, a recent transcranial magnetic stimulation (TMS) study [52] demonstrates that TMS over the right posterior parietal cortex (PPC) during a top-down selection by color diminished top-down control for the left hemifield while enhancing this for the right hemifield. In contrast, TMS over the left PPC does not change the pattern of performance. On the one hand, the results underline a strong interaction between spatial and nonspatial aspects of visual selection within the FPN [53]. Yet, on the other hand, the laterality and hemifield specificity within a subregion of the FPN is evident. Future work could apply TMS on single left and right subregions of the FPN to determine their selective role during different subprocesses of attention in the left and right hemifields, respectively.

### Conclusions and Perspectives

In summary, our data show that spatial coding within the dorsal frontoparietal network is dynamic. The two factors which may account for the changes in spatial coding appear to be the component of attention (endogenous vs. exogenous) and the dynamic of the focus of attention (stationary vs. shifting). The changes in contralateral preference emphasize the complexity of spatial representations in the human brain and may lead to further clarification of the current discussion of asymmetries in spatial attention and of pathophysiological models of hemispatial neglect. Firstly, our results give rise to a possible solution to the ongoing debate about spatial coding in the dorsal path of the attention system as described in the introduction [9]–[13], [54]. In these experiments, different paradigms were used that varied in addition to other factors in the subprocess of attention (spatial orienting/working memory/attentional shifting tasks) and in the type of attention (endogenous/exogenous; overt/covert). As we could show the dependency of the pattern of contralateral preference on the subprocess of attention, this may account for the conflicting results in recent attention research. However, a recent study demonstrates that contralateral preference in some cortical regions changes between finger pointing and saccade tasks [14], i.e. different response modalities. Thus, further research is needed to clarify in which regions changes in spatial coding largely depend on different attention subprocesses or different response modalities. Secondly, our results contribute to the discussion of models for spatial neglect. The pathophysiology of spatial neglect is currently under discussion since current models do not account for the broad variation in the clinical syndrome [4], [6], [7]. It is possible that several distinct disorders or cognitive processes have been erroneously pooled under the single label “spatial neglect”. For instance, the two cardinal diagnostic tests – line bisection and search/line cancellation – demonstrate the heterogeneity of the disorder, with double dissociations between patients and tests, as well as differences in neglect lesion localization [7], [55]–[57]. Our data support the view of a more complex pathophysiology of this syndrome and suggest a more detailed exploration of patients with circumscribed lesions using paradigms that allow differentiation between distinct subprocesses of spatial attention, as well as between other cognitive processes [7], [58], e.g. spatial working memory.

## References

[1] Danckert JA, Goodale MA, Johnson-Frey SH (2003). Ups and Downs in the Visual Control of Actions.. Taking Action: Cognitive Neuroscience Perspectives onIntentional Actions.

[2] Hemond CC, Kanwisher NG, Op de Beeck HP (2007). A preference for contralateral stimuli in human object- and face-selective cortex.. PLoS ONE.

[3] Niemeier M, Goltz HC, Kuchinad A, Tweed DB, Vilis T (2005). A contralateral preference in the lateral occipital area: sensory and attentional mechanisms.. Cereb Cortex.

[4] Mesulam MM (1999). Spatial attention and neglect: parietal, frontal and cingulate contributions to the mental representation and attentional targeting of salient extrapersonal events.. Philos Trans R Soc Lond B Biol Sci.

[5] Corbetta M, Shulman GL (2002). Control of goal-directed and stimulus-driven attention in the brain.. Nat Rev Neurosci.

[6] Corbetta M, Kincade MJ, Lewis C, Snyder AZ, Sapir A (2005). Neural basis and recovery of spatial attention deficits in spatial neglect.. Nat Neurosci.

[7] Milner AD, McIntosh RD (2005). The neurological basis of visual neglect.. Curr Opin Neurol.

[8] Corbetta M, Kincade JM, Shulman GL (2002). Neural systems for visual orienting and their relationships to spatial working memory.. J Cogn Neurosci.

[9] Vandenberghe R, Geeraerts S, Molenberghs P, Lafosse C, Vandenbulcke M, Peeters K, Peeters R, Van HP, Orban GA (2005). Attentional responses to unattended stimuli in human parietal cortex.. Brain.

[10] Schwartz S, Vuilleumier P, Hutton C, Maravita A, Dolan RJ (2005). Attentional load and sensory competition in human vision: modulation of fMRI responses by load at fixation during task-irrelevant stimulation in the peripheral visual field.. Cereb Cortex.

[11] Sereno MI, Pitzalis S, Martinez A (2001). Mapping of contralateral space in retinotopic coordinates by a parietal cortical area in humans.. Science.

[12] Hagler DJ, Sereno MI (2006). Spatial maps in frontal and prefrontal cortex.. Neuroimage.

[13] Silver MA, Ress D, Heeger DJ (2005). Topographic maps of visual spatial attention in human parietal cortex.. J Neurophysiol.

[14] Hagler DJ, Riecke L, Sereno MI (2007). Parietal and superior frontal visuospatial maps activated by pointing and saccades.. Neuroimage.

[15] Muller NG, Bartelt OA, Donner TH, Villringer A, Brandt SA (2003). A physiological correlate of the “Zoom Lens” of visual attention.. J Neurosci.

[16] Muller NG, Donner TH, Bartelt OA, Brandt SA, Villringer A, Kleinschmidt A (2003). The functional neuroanatomy of visual conjunction search: a parametric fMRI study.. Neuroimage.

[17] Donner T, Kettermann A, Diesch E, Ostendorf F, Villringer A, Brandt SA (2000). Involvement of the human frontal eye field and multiple parietal areas in covert visual selection during conjunction search.. Eur J Neurosci.

[18] Gitelman DR (2002). ILAB: a programm for postexperimental eye movement analysis.. Behav Res Methods Instrum Comput.

[19] Turatto M, Benso F, Facoetti A, Galfano G, Mascetti GG, Umilta C (2000). Automatic and voluntary focusing of attention.. Percept Psychophys.

[20] Kraft A, Muller NG, Hagendorf H, Schira MM, Dick S (2005). Interactions between task difficulty and hemispheric distribution of attended locations: implications for the splitting attention debate.. Brain Res Cogn Brain Res.

[21] Kraft A, Pape N, Hagendorf H, Schmidt S, Naito A (2007). What determines sustained visual attention? The impact of distracter positions, task difficulty and visual fields compared.. Brain Res.

[22] Forster DH, Ward PA (1991). Asymmetries in oriented-line detection indicate two orthogonal filters in early vision.. Proc R Soc Lond B Biol Sci.

[23] Talairach J, Tournoux P (1988). Co-planar Stereotactic Atlas of the Human Brain.

[24] Donner TH, Kettermann A, Diesch E, Ostendorf F, Villringer A (2002). Visual feature and conjunction searches of equal difficulty engage only partially overlapping frontoparietal networks.. Neuroimage.

[25] Beauchamp MS, Petit L, Ellmore TM, Ingeholm J, Haxby JV (2001). A parametric fMRI study of overt and covert shifts of visuospatial attention.. Neuroimage.

[26] Haynes JD, Rees G (2005). Predicting the stream of consciousness from activity in human visual cortex.. Curr Biol.

[27] MacMillan NACCD (2004). Detection Theory: A User's Guide.

[28] Zenger B, Fahle M (1997). Missed targets are more frequent than false alarms: a model for error rates in visual search.. J Exp Psychol Hum Percept Perform.

[29] Kastner S, Pinsk MA (2004). Visual attention as a multilevel selection process.. Cogn Affect Behav Neurosci.

[30] Corbetta M, Miezin FM, Shulman GL, Petersen SE (1993). A PET study of visuospatial attention.. J Neurosci.

[31] Macaluso E, Patria F (2007). Spatial re-orienting of visual attention along the horizontal or the vertical axis.. Exp Brain Res.

[32] Nakayama K, Mackeben M (1989). Sustained and transient components of focal visual attention.. Vision Res.

[33] Weichselgartner E, Sperling G (1987). Dynamics of automatic and controlled visual attention.. Science.

[34] Collie A, Maruff P, Yucel M, Danckert J, Currie J (2000). Spatiotemporal distribution of facilitation and inhibition of return arising from the reflexive orienting of covert attention.. J Exp Psychol Hum Percept Perform.

[35] Wolfe JM, Butcher SJ, Lee C, Hyle M (2003). Changing your mind: on the contributions of top-down and bottom-up guidance in visual search for feature singletons.. J Exp Psychol Hum Percept Perform.

[36] Yantis S, Schwarzbach J, Serences JT, Carlson RL, Steinmetz MA, Pekar JJ, Courtney SM (2002). Transient neural activity in human parietal cortex during spatial attention shifts.. Nat Neurosci.

[37] Kincade JM, Abrams RA, Astafiev SV, Shulman GL, Corbetta M (2005). An event-related functional magnetic resonance imaging study of voluntary and stimulus-driven orienting of attention.. J Neurosci.

[38] Corbetta M, Kincade JM, Ollinger JM, McAvoy MP, Shulman GL (2000). Voluntary orienting is dissociated from target detection in human posterior parietal cortex.. Nat Neurosci.

[39] Hopfinger JB, Buonocore MH, Mangun GR (2000). The neural mechanisms of top-down attentional control.. Nat Neurosci.

[40] Robertson LC, Lamb MR, Knight RT (1988). Effects of lesions of temporal-parietal junction on perceptual and attentional processing in humans.. J Neurosci.

[41] Vallar G, Bisiach E, Cerizza M, Rusconi ML (1988). The role of the left hemisphere in decision-making.. Cortex.

[42] Hahn B, Ross TJ, Stein EA (2006). Neuroanatomical dissociation between bottom-up and top-down processes of visuospatial selective attention.. Neuroimage.

[43] Nobre AC, Sebestyen GN, Gitelman DR, Mesulam MM, Frackowiak RS (1997). Functional localization of the system for visuospatial attention using positron emission tomography.. Brain.

[44] Schluter ND, Krams M, Rushworth MF, Passingham RE (2001). Cerebral dominance for action in the human brain: the selection of actions.. Neuropsychologia.

[45] Kim YH, Gitelman DR, Nobre AC, Parrish TB, LaBar KS (1999). The large-scale neural network for spatial attention displays multifunctional overlap but differential asymmetry.. Neuroimage.

[46] Peelen MV, Heslenfeld DJ, Theeuwes J (2004). Endogenous and exogenous attention shifts are mediated by the same large-scale neural network.. Neuroimage.

[47] Mayer AR, Dorflinger JM, Rao SM, Seidenberg M (2004). Neural networks underlying endogenous and exogenous visual-spatial orienting.. Neuroimage.

[48] Mort DJ, Perry RJ, Mannan SK, Hodgson TL, Anderson E, Quest R (2003). Differential cortical activation during voluntary and reflexive saccades in man.. Neuroimage.

[49] Gainotti G (1996). Lateralization of brain mechanisms underlying automatic and controlled forms of spatial orienting of attention.. Neurosci Biobehav Rev.

[50] Coull JT, Frith CD (1998). Differential activation of right superior parietal cortex and intraparietal sulcus by spatial and nonspatial attention.. Neuroimage.

[51] Sereno AB, Maunsell JH (1998). Shape selectivity in primate lateral intraparietal cortex.. Nature.

[52] Hung J, Driver J, Walsh V (2005). Visual selection and posterior parietal cortex: effects of repetitive transcranial magnetic stimulation on partial report analyzed by Bundesen's theory of visual attention.. J Neurosci.

[53] Tsal Y, Lavie N (1988). Attending to color and shape: the special role of location in selective visual processing.. Percept Psychophys.

[54] Schluppeck D, Glimcher P, Heeger DJ (2005). Topographic organization for delayed saccades in human posterior parietal cortex.. Journal of Neurophysiology.

[55] Heilman KM, Watson RT, Valenstein E, Damasio AR, Kertesz A (1983). Localization of lesions in neglect.. Localization in neuropsychology.

[56] Rorden C, Karnath HO (2004). Using human brain lesions to infer function: a relic from a past era in the fMRI age?. Nat Rev Neurosci.

[57] Ferber S, Karnath HO (2001). How to assess spatial neglect–line bisection or cancellation tasks?. J Clin Exp Neuropsychol.

[58] Duncan J, Bundesen C, Olson A, Humphreys G, Chavda S (1999). Systematic analysis of deficits in visual attention.. J Exp Psychol Gen.

